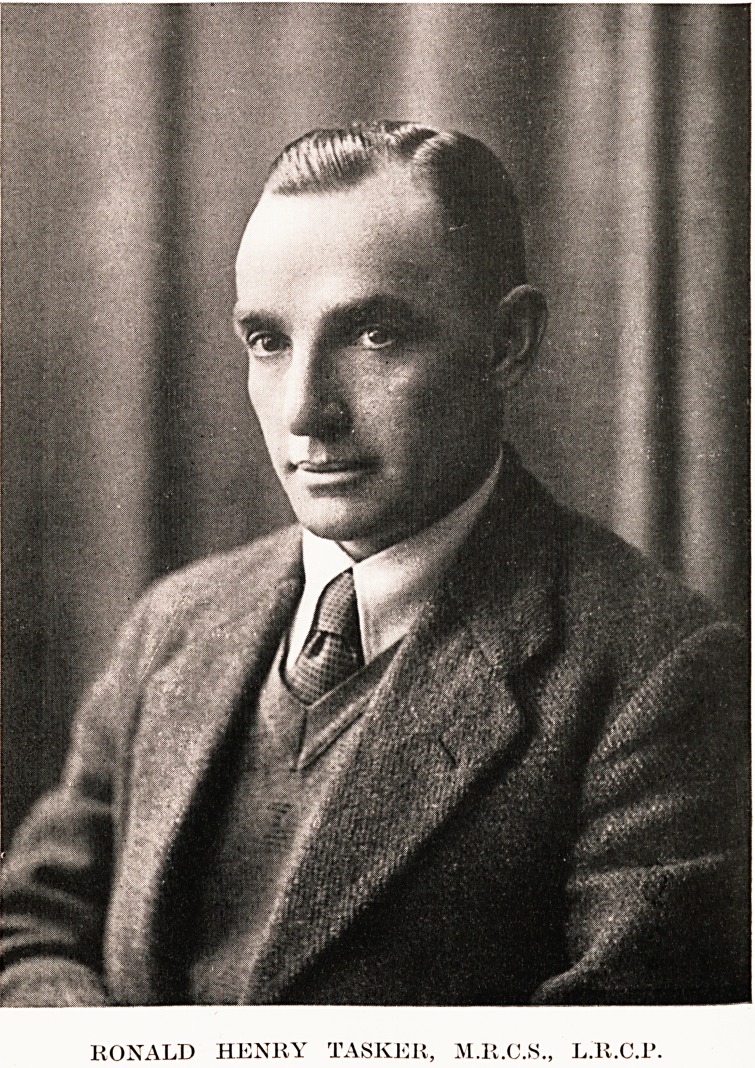# Ronald Henry Tasker

**Published:** 1943

**Authors:** 


RONALD HENRY TASKER, M.R.C.S., L.R.C.P.

### RONALD HENRY TASKER, M.R.C.S., L.R.C.P.

THE tragic death of Ronald Henry Tasker, in November, 1942, will be felt as a deep personal loss to many old students of the Bristol Medical School where he began his training in 1909. He attended the clinical practice of the Bristol General Hospital, to which he was later House Surgeon and House Physician, and served during the last war as Temporary Surg. Lieut., R.N., in H.M.S. *Centurion*. After leaving the Hospital he held resident appointments at Herefordshire General Hospital, Taunton Hospital, and Ham Green Hospital, Bristol. In 1926 he became Medical Officer to the Burmah Oil Company, for whom he worked enthusiastically and well for sixteen years. The Japanese invasion found him in charge of the Company's hospital in Syriam, Rangoon, and he was greatly saddened by witnessing the destruction of the station and by sustaining the loss of his valuable collection of books, cuttings, and stamps, together with all his personal belongings. After the demolitions were completed he made an adventurous escape to India, finishing the remainder of his term of office in Assam. He was on his way home when his ship was torpedoed in the South Atlantic. With 53 others Tasker was adrift in a lifeboat for many days, and only two survived the ordeal. The sole survivor to reach England, the other being now a prisoner of war, has told the grim story of the events resulting in his untimely death.

In 1930 he married Miss Dorothy Buckmaster, M.B., B.S., daughter of the late Professor of Physiology of Bristol University, and he leaves her with a young son and daughter. The deepest sympathy is extended to his wife in her overwhelming loss.

Ronald Tasker's joyful personality was many sided, but in everything he undertook he was meticulous, keen, skilful, and loyal. All his professional work was characterized by great ability and carefulness, and Burma afforded him ample opportunities for using his surgical skill. The homage he always paid to his teaching school and to those who were his mentors inspired his work, while attention to detail, clinical honesty and acumen rendered him a very worthy member of his profession.

He was very widely read and even while a student he had begun to build up a fine library, in which his favourite subjects, anthropology and comparative religion, were well represented.

Yet to very many, both in this country and in the East, his name will ever be remembered for his prowess in athletics. From boyhood and until the year of his death he was an ardent and active sportsman. He played for the University in hockey and association football, was a county player in football, tennis, and badminton, and in his student days played, as an amateur, for Bristol City, then in the second division of the Football League. While playing games with skill and utter enjoyment, he studied them with the same care as he would give to a scientific problem. His opponents knew him as a relentless but just adversary, popular with all.

Vital, companionable, abounding in energy and high spirits—to have made Tasker's acquaintance was a real pleasure, to have known him well was the privilege of a lifetime. By his innate good nature and sincerity he enjoyed the affection and regard of his fellow men in widely different spheres of life. His sad fate will leave a gap in the lives of his friends which will never be filled.

## Figures and Tables

**Figure f1:**